# Supplementary material for: Aclarubicin stimulates RNA polymerase II elongation at closely spaced divergent promoters
Source: bioRxiv. 2023 Jan 10:2023.01.09.523323. Preprint. [Version 1] doi: 10.1101/2023.01.09.523323 (PMC9882078; doi:10.1101/2023.01.09.523323)
Supplement: Supplement 1 — Fig. S1: Impacts of drug treatment after 1 hour and 24 hours (A) Percent of cells with detectable nucleolus after 1 hour of treatment and 24 hours treatment. (B) Percent of cells with detectable cleaved-caspase (cell death) staining after 1 hour of treatment and 24 hours treatment (C) Cell size measurements after 1 hr of treatment and 24hrs treatment. (D) Immunofluorescent images of DAPI, Cleaved-caspase (CC) and Fibrillarin (nucleolar marker) after 24 hours treatment. (E) Example of cleaved caspase-positive cell. Scale bar = 5µm(A) Immunofluorescent images of DAPI, Cleaved-caspase (CC) and Fibrillarin (nucleolar marker) for cells at 24 hr drug treatment. Scale bar = 5µm. ns = not significant, **P<0.01; **** P<0.0001. Ordinary one-way ANOVA with multiple comparisons. Fig. S2: Drug treatment impacts the relative distribution of RNA Pol II Ser5P and Ser2P (A) Heatmap aligned to the transcriptional start site (TSS) of all promoters showing normalized counts of RNA Pol II ser5P CUT&Tag signal clustered via k-means clustering derived from CUTAC datasets (k=3). (B) Enlarged comparison of accessibility differences between different drug groups and controls. Gray box marks upstream promoter region. (C) Heatmap aligned to TSS of all promoters showing normalized counts of RNA Pol II Ser2P CUT&Tag signal clustered via k-means clustering (k=3) derived from CUTAC datasets. (D) Enlarged comparison of G-quadruplex differences between different drug groups and controls. Gray box marks upstream promoter region. (E) Representative UCSC browser track snapshot of RNA Pol II ser5P and RNA Pol II Ser2P distribution at Cluster I gene. (F) Representative UCSC browser track snapshot of RNA Pol II ser5P and RNA Pol II Ser2P distribution at Cluster II gene. Fig. S3: Promoter distance and orientation effects RNA Pol II, G-quadruplexes, accessibility, and torsion. Heatmaps of CUT&Tag data targeting RNA Pol II ser5P (A) RNA Pol II ser2P (B) and G-quadruplexes (C) aligned to the transcriptional s [file NIHPP2023.01.09.523323v1-supplement-1.pdf]

bioRxiv preprint doi: https://doi.org/10.1101/2023.01.09.523323; this version posted January 10, 2023. The copyright holder for this preprint (which was not certified by peer review) is the author/funder, who has granted bioRxiv a license to display the preprint in perpetuity. It is made available under aCC-BY 4.0 International license.

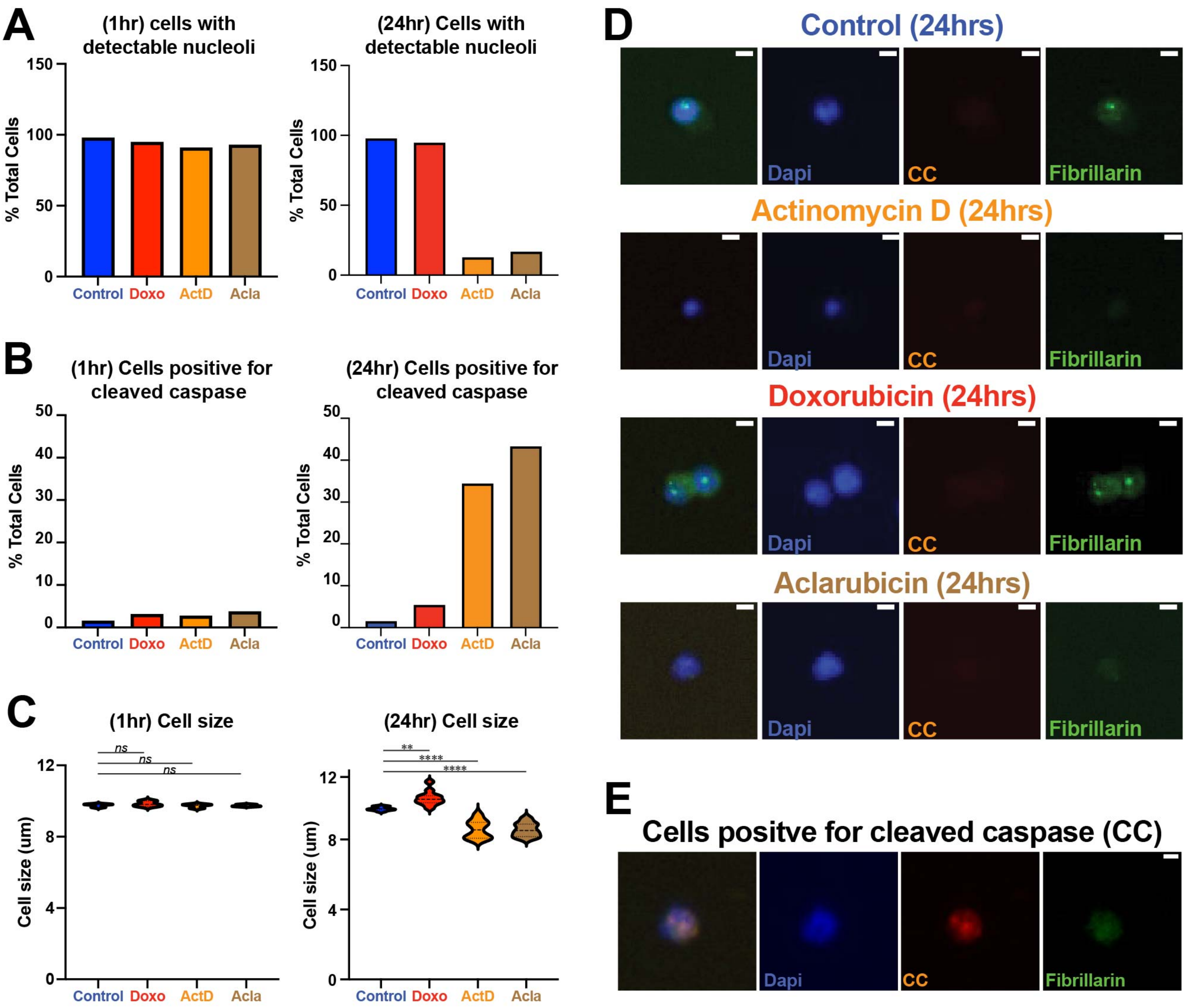

Fig. S1: Impacts of drug treatment after 1 hour and 24 hours (**A**) Percent of cells with detectable nucleolus after 1 hour of treatment and 24 hours treatment. (**B**) Percent of cells with detectable cleaved-caspase (cell death) staining after 1 hour of treatment and 24 hours treatment (**C**) Cell size measurements after 1 hr of treatment and 24hrs treatment. (**D**) Immunofluorescent images of DAPI, Cleaved-caspase (CC) and Fibrillarin (nucleolar marker) after 24 hours treatment. (**E**) Example of cleaved caspase-positive cell. Scale bar = 5µm(A) Immunofluorescent images of DAPI, Cleaved-caspase (CC) and Fibrillarin (nucleolar marker) for cells at 24 hr drug treatment. Scale bar = 5µm. *ns* = not significant, **P<0.01; **** P<0.0001. Ordinary one-way ANOVA with multiple comparisons.

bioRxiv preprint doi: https://doi.org/10.1101/2023.01.09.523323; this version posted January 10, 2023. The copyright holder for this preprint (which was not certified by peer review) is the author/funder, who has granted bioRxiv a license to display the preprint in perpetuity. It is made available under aCC-BY 4.0 International license.

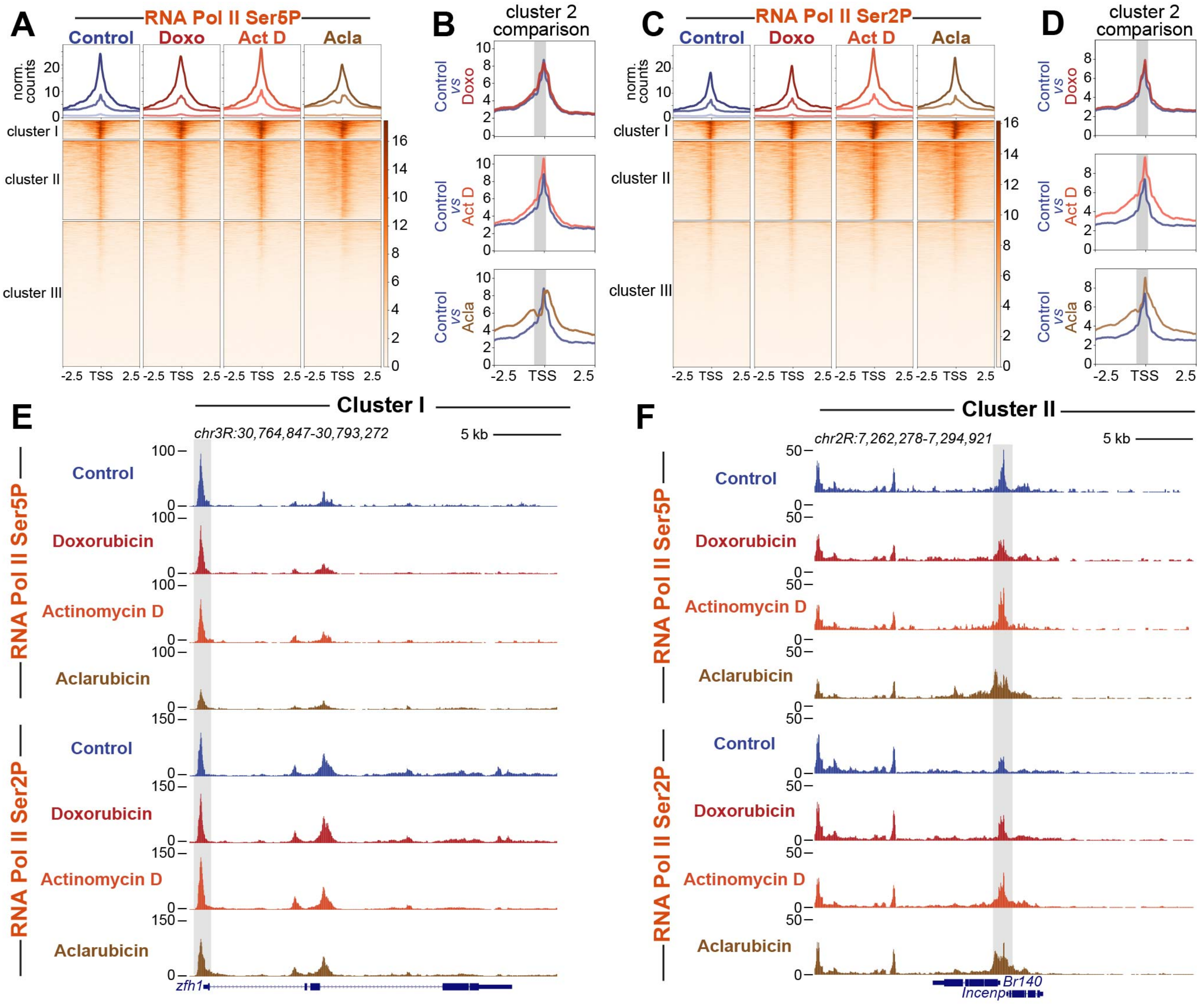

Fig. S2: Drug treatment impacts the relative distribution of RNA Pol II Ser5P and Ser2P (**A**) Heatmap aligned to the transcriptional start site (TSS) of all promoters showing normalized counts of RNA Pol II ser5P CUT&Tag signal clustered via k-means clustering derived from CUTAC datasets (k=3). (**B**) Enlarged comparison of accessibility differences between different drug groups and controls. Gray box marks upstream promoter region. (**C**) Heatmap aligned to TSS of all promoters showing normalized counts of RNA Pol II Ser2P CUT&Tag signal clustered via k-means clustering (k=3) derived from CUTAC datasets. (**D**) Enlarged comparison of G-quadruplex differences between different drug groups and controls. Gray box marks upstream promoter region. (**E**) Representative UCSC browser track snapshot of RNA Pol II ser5P and RNA Pol II Ser2P distribution at Cluster I gene. (**F**) Representative UCSC browser track snapshot of RNA Pol II ser5P and RNA Pol II Ser2P distribution at Cluster II gene.

bioRxiv preprint doi: https://doi.org/10.1101/2023.01.09.523323; this version posted January 10, 2023. The copyright holder for this preprint (which was not certified by peer review) is the author/funder, who has granted bioRxiv a license to display the preprint in perpetuity. It is made available under aCC-BY 4.0 International license.

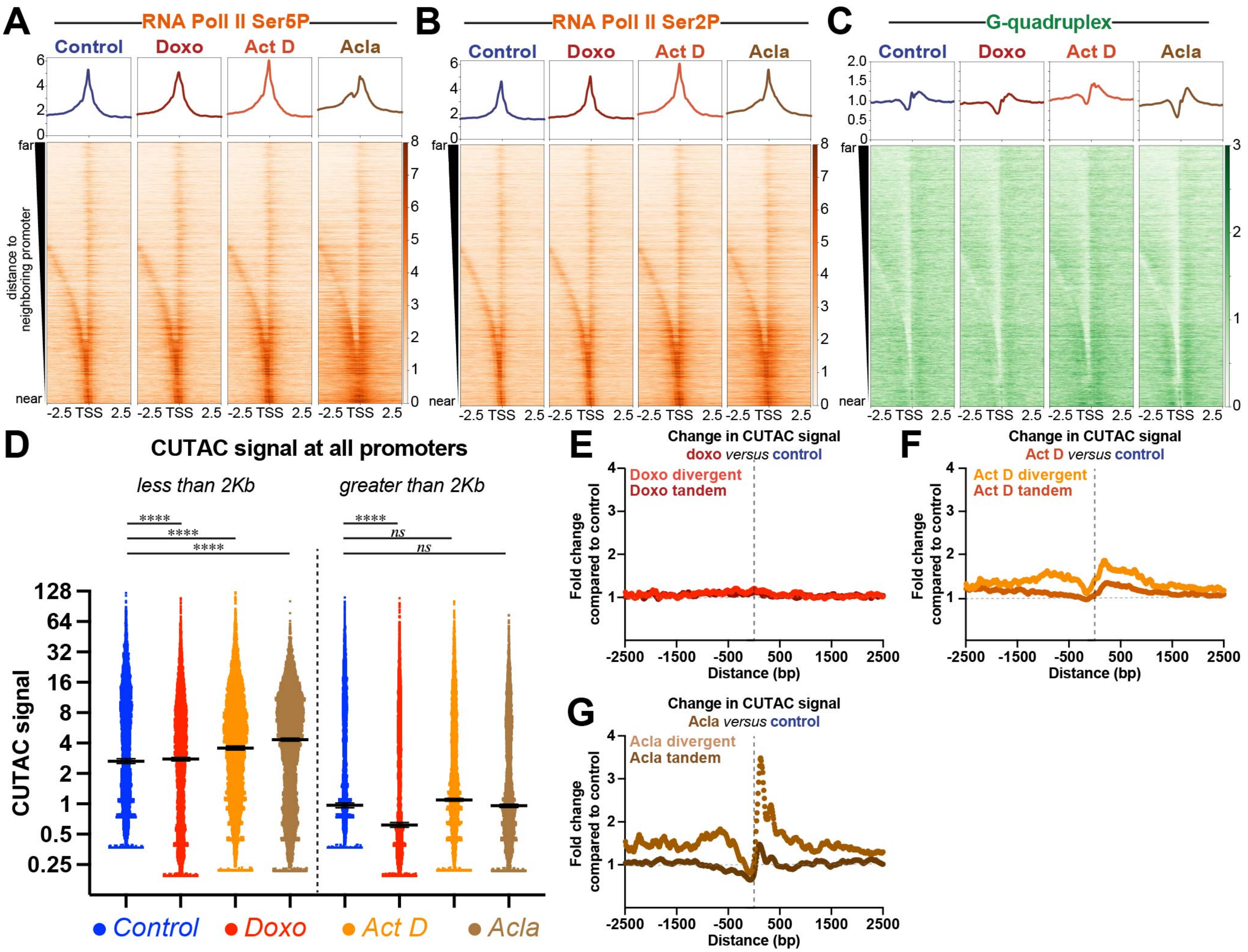

Fig. S3: Promoter distance and orientation effects RNA Pol II, G-quadruplexes, accessibility, and torsion. Heatmaps of CUT&Tag data targeting RNA Pol II ser5P (**A**) RNA Pol II ser2P (**B**) and G-quadruplexes (**C**) aligned to the transcriptional start site (TSS) of all promoters sorted by distance to nearest upstream promoter element and plotted in descending order. (**D**) Plot showing CUTAC chromatin accessibility signal for promoters less than 2kb apart and greater than 2kb apart. Median shown as black bar with 95% confidence interval. (**E**) Plot showing fold change of CUTAC chromatin accessibility centered on the TSS for doxorubicin *vs* control (E), actinomycin D *vs* control (**F**) and aclarubicin *vs* control (**G**) Horizontal dotted line indicates 1-fold. Vertical dotted line indicates TSS. (**H**) Plot showing TMP-seq signal for promoters less than 2kb apart and greater than 2kb apart. **** = p< 0.0001 Kruskal-Wallis test with multiple comparisons for panel D.

bioRxiv preprint doi: https://doi.org/10.1101/2023.01.09.523323; this version posted January 10, 2023. The copyright holder for this preprint (which was not certified by peer review) is the author/funder, who has granted bioRxiv a license to display the preprint in perpetuity. It is made available under aCC-BY 4.0 International license.

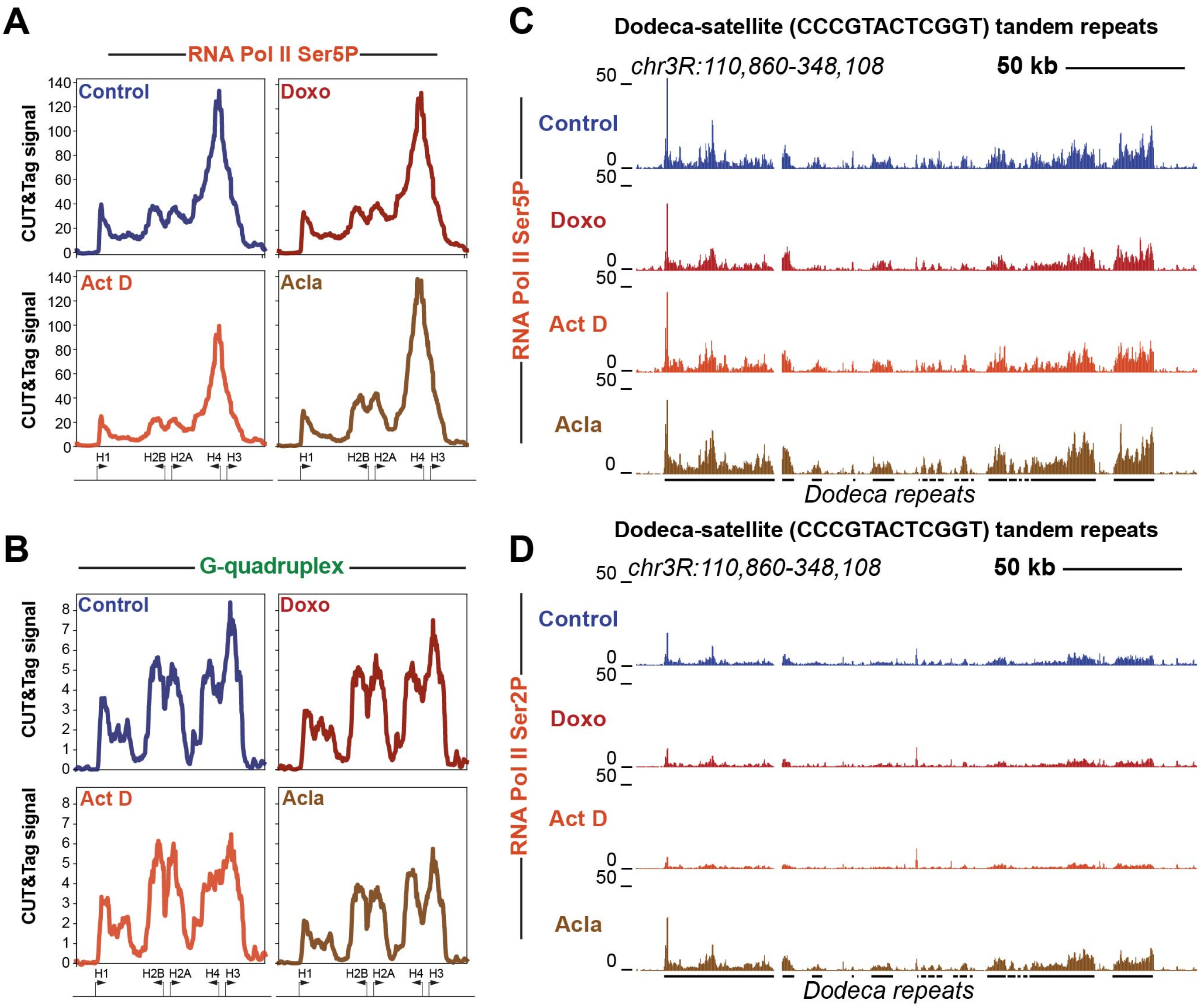

Fig. S4: Histone cluster and Dodeca-satellite repeats show distinct responses to drug treatment (**A**) Average coverage plot of histone clusters showing CUT&Tag data targeting RNA Pol II ser5P. (**B**) Average coverage plot of histone clusters showing CUT&Tag data targeting G-quadruplexes. Arrows at the bottom of average plots indicate approximate positions of histone genes. (**C**) UCSC browser track snapshot of CUT&Tag data targeting RNA Pol II ser5P at Dodeca-satellite repeats. (**D**) UCSC browser track snapshot of CUT&Tag data targeting RNA Pol II ser2P at Dodeca-satellite repeats. Black lines below the browser tracks indicate location of Dodeca-satellite repeats.
